# Mutations in the gene of the Gα subunit of the heterotrimeric G protein are the cause for the *brachytic1* semi-dwarf phenotype in barley and applicable for practical breeding

**DOI:** 10.1186/s41065-017-0045-1

**Published:** 2017-09-05

**Authors:** Ilka Braumann, Christoph Dockter, Sebastian Beier, Axel Himmelbach, Finn Lok, Udda Lundqvist, Birgitte Skadhauge, Nils Stein, Shakhira Zakhrabekova, Ruonan Zhou, Mats Hansson

**Affiliations:** 1 Carlsberg Research Laboratory, J. C. Jacobsens Gade 4, DK-1799 Copenhagen V, Denmark; 2 Leibniz Institute of Plant Genetics and Crop Plant Research (IPK), OT Gatersleben, DE-06466 Stadt Seeland, Germany; 3 Nordic Genetic Resource Center (NordGen), Smedjevägen 3, SE-23053 Alnarp, Sweden; 4 Department of Biology, Lund University, Sölvegatan 35, SE-22362 Lund, Sweden

**Keywords:** *Ari-i*, *Ari-m*, *Brh1*, *Hordeum vulgare*, Semi-dwarf

## Abstract

**Background:**

Short-culm mutants have been widely used in breeding programs to increase lodging resistance. In barley (*Hordeum vulgare* L.), several hundreds of short-culm mutants have been isolated over the years. The objective of the present study was to identify the *Brachytic1* (*Brh1*) semi-dwarfing gene and to test its effect on yield and malting quality.

**Results:**

Double-haploid lines generated through a cross between a *brh1.a* mutant and the European elite malting cultivar Quench, showed good malting quality but a decrease in yield. Especially the activities of the starch degrading enzymes β-amylase and free limit dextrinase were high. A syntenic approach comparing markers in barley to those in rice (*Oryza sativa* L.), sorghum (*Sorghum bicolor* Moench) and brachypodium (*Brachypodium distachyon* P. Beauv) helped us to identify *Brh1* as an orthologue of rice *D1* encoding the Gα subunit of a heterotrimeric G protein. We demonstrated that *Brh1* is allelic to *Ari-m*. Sixteen different mutant alleles were described at the DNA level.

**Conclusions:**

Mutants in the *Brh1* locus are deficient in the Gα subunit of a heterotrimeric G protein, which shows that heterotrimeric G proteins are important regulators of culm length in barley. Mutant alleles do not have any major negative effects on malting quality.

**Electronic supplementary material:**

The online version of this article (10.1186/s41065-017-0045-1) contains supplementary material, which is available to authorized users.

## Background

Semi-dwarfism is regarded as a valuable trait in cereal breeding, as cultivars with a short stature show increased lodging resistance [6, 25]. Due to the potential agronomic usefulness, many different types of semi-dwarf mutants have been identified in barley (*Hordeum vulgare*) during the last 70 years. These semi-dwarf mutants have obtained different names since they often have other pleiotropic characters which might have been in focus of the involved research group. The major semi-dwarf subgroups are *Brachytic* (*Brh*), *Breviaristatum* (*Ari*), *Slender dwarf* (*Sld*), *Erectoides* (*Ert*), *Semidwarf* (*Sdw*), *Semi-brachytic* (*Uzu*) and *Dense spike* (*Dsp*) [10]. However, only a few barley semi-dwarfing genes have successfully been applied in breeding programs – The *uzu1.a* allele was one of the first short-culm alleles to be used [31] and has been introduced in almost all Japanese hull-less barley cultivars [26]; two different alleles of the *Semidwarf 1* gene, *sdw1.c* (originally named *denso*) and *sdw1.d*, are most likely the dominating short-culm alleles in the today’s European elite cultivars [10]; *ert-k.32* was isolated after X-ray treatment in the cultivar Bonus and released as the cultivar Pallas [13]; *ari-e.GP* was isolated after γ-ray mutagenesis of cultivar Maythorpe in 1956 and released as Golden Promise [1, 12]; *sdw4.ba* was isolated after γ-ray treatment of the Chinese cultivar Zhenongguangmangerleng in the late 1960s and is now probably widespread in many semi-dwarf barley accessions in China through the cultivar released as Zhepi 1 [37, 38].

Plant hormones like gibberellic acid (GA) and brassinosteroids (BR) are the major regulators of plant height. It is therefore not surprising that successful “Green revolution” semi-dwarfing genes can be exemplified by *Reduced height1* (*Rht1*) in wheat encoding a transcriptional regulator with a DELLA domain involved in GA signaling [23], *Semi-dwarf1* (*Sd1*) in rice [3, 28] and *Sdw1* in barley [36] both deficient in a gibberellin 20-oxidase of the GA biosynthetic pathway, and *Uzu1* in barley encoding the brassinosteroid receptor [7, 9].

**Fig. 1:**
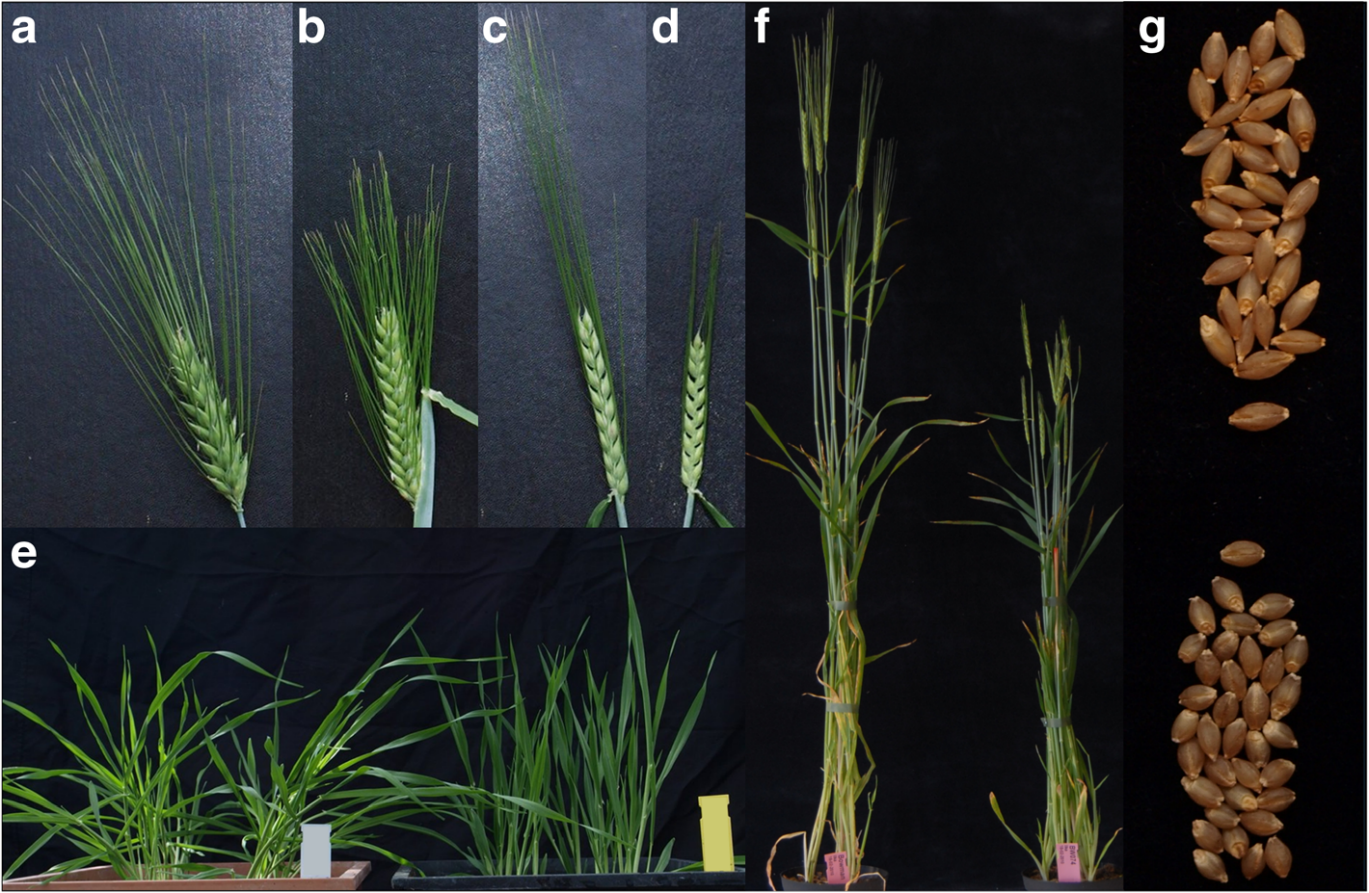
Phenotype of *brh1* mutants in barley compared to the respective mother cultivar. (**a**) Awn length of cultivar Steptoe, (**b**) *brh1.ae*, (**c**) cultivar Bowman, and (**d**) near-isogenic line BW074 (*brh1.a*). **e**. The erectness of seedlings of cultivar Bowman (left) and BW074 (*brh1.a*). **f**. Overall size of Bowman and BW074 (*brh1.a*) after heading. **g**. Global-shaped kernels of *brh1.a* (bottom) compared to cultivar Himalaya (top)

An interesting mutant, not only in the context of plant breeding but also for identification of genetic factors determining plant height, is *brachytic1* (*brh1*), which was originally identified after a spontaneous mutation event in the barley cultivar Himalaya [24, 30]. Outgrown plants have an upright gross morphology with short leaves, culms, spikes, awns and kernels, and their seedling leaf is reduced in length (Fig. 1). The mutant phenotype is easy to classify at all stages of growth (www.nordgen.org/bgs). In total, ten recessive *brh1* alleles have been isolated in a wide range of different genetic backgrounds. One allele was previously classified as *ari-i* but shown to be allelic to *brh1* through diallelic crosses [33] (Table 1). *Brh1* was mapped to chromosome 7HS and is inherited in a monofactorial recessive manner [24, 30]. Eight of the ten *brh1* alleles are further available as near-isogenic lines through recurrent backcrosses to the two-row spring barley cultivar Bowman (Table 1) [11]. Trait analysis of *brh1* mutants in the genetic background of Bowman showed that several *brh1* alleles reduce lodging without causing significant yield reduction [8]. In addition, the globe-shaped form of *brh1* mutant seeds could also be of potential value; such seed form is generally preferred by maltsters as it is associated with an even germination in the malt house. However, the overall effect of *brh1* mutations on malting is currently unknown. Still, when elucidating if *brh1* alleles could indeed serve as an alternative dwarfing gene in barley, it is of particular importance to exclude a negative impact of the mutation on malting quality.

**Table 1:** Known brh1 mutant alleles, their genetic background, their availability as Bowman near-isogenic lines, and introgression regions on chromosome 7H

Allele	Genetic background	Bowman near-isogenic line	Introgression region on 7HS (cM)
*ari-i.38* ^b^	Bonus	BW047^b^	0 – 13.19
*brh1.a* ^a^	Himalaya	BW074^b^	0 – 13.19
*brh1.aa* ^*d*^	Aapo	BW075^b^	0 – 48.45
*brh1.ae* ^*c*^	Steptoe	BW076^b^	0 – 20.56
*brh1.c* ^a^	Moravian	no line	-
*brh1.e* ^a^	Aramir	BW077^b^	0 – 13.19
*brh1.f* ^a^	Domen	no line	-
*brh1.t* ^a^	Akashinriki	BW078^b^	8.77 – 26
*brh1.x* ^a^	Volla	BW079^b^	0 – 8.77
*brh1.z* ^d^	Aapo	BW080^b^	0 – 32.35

The near-isogenic lines have been prepared previously as well as the determination of the introgressions [11]

Source of seed material: ^a^NSGC, National Small Grains Collection, U.S. Department of Agriculture - Agricultural Research Service, Aberdeen, Idaho, USA; ^b^Nordic Genetic Resource Center, Alnarp, Sweden; ^c^Andris Kleinhofs, Washington State University, Pullman, WA, USA; ^d^not available

The aim of the present study was to identify the *Brh1* semi-dwarfing gene and to test its effect on yield and malting quality. We show that *Brh1* encodes a subunit of a heterotrimeric G protein and mutant alleles of *Brh1* do not have any major negative effects on malting quality. Heterotrimeric G proteins transfer signals from receptors (G-protein-coupled receptors) to downstream enzymes (effectors) in signaling cascades [32]. The heterotrimeric G protein complex consists of one Gα, one Gβ and one Gγ subunit. *Brh1* encodes a Gα subunit. This is especially interesting in the view that it was recently shown that barley *Ari-e* encodes a Gγ subunit [35], being orthologous to *DEP1* in rice [16]. In addition, barley *Brh2* encodes a U-box E3 ubiquitin ligase, orthologous to *TUD1* in rice (Braumann et al. unpublished). TUD1 has been suggested to physically interact with the Gα subunit in rice [15]. Thus, within a short period of time, heterotrimeric G proteins have been identified as a key regulator of plant architecture in barley.

## Methods

### Plant material

All mutant lines were obtained from public non-profit organizations or individual contributors: *brh1.a*, *brh1.e*, *brh1.t*, *brh1.x*, *brh1.c* and *brh1.f* from the National Small Grains Collection (U.S. Department of Agriculture - Agricultural Research Service, Aberdeen, Idaho, USA); *brh1.ae* from Andris Kleinhofs, (Washington State University, Pullman, WA, USA); *ari-i*.38 and the eight Bowman near-isogenic lines BW047 (*ari-i.38*), BW074 (*brh1a*), BW075 (*brh1.aa*), BW076 (*brh1.ae*), BW077 (*brh1.e*), BW078 (*brh1.t*), BW079 (*brh1.x*) and BW080 (*brh1.z*) from the Nordic Genetic Resource Center (NordGen, Alnarp, Sweden).

### Phenotypic description of mutant and wild type lines

For phenotypic descriptions, plants were grown in the greenhouse under 16-h-light / 8-h-dark cycles. The culm length measured is the distance between the soil and the collar of the spike. Further, the distances from the collar of the spike to the tip of the spike and to the tip of the longest awn was measured. Awn length was normalized to the length of the spike by forming the ratio of [length from spike collar to tip of longest awn] / [length from spike collar to tip of spike]. Per line 12 plants were grown in large square formed pots. The significance of differences in culm length and awn length between mutant lines and respective wild types was tested by a two sided t-test for two independent populations with equal variance assumption in Microsoft Excel. The number of biological replicates was *n* = 3 (in the case of BW047) to *n* = 12 depending on the number of seeds that germinated per line.

### Generation of double-haploid plants

In order to transfer *brh1.a* into a more modern genetic background we performed crosses between the Bowman near-isogenic line BW074 (*brh1.a*) and the European elite malting cultivar Quench. F_1_ seeds were subjected to embryo rescue 25 days after fertilization. The outer parts of the husk of the developing grains were removed and the grains were sterilized in 4% sodium hypochlorite for 10 min followed by rinsing in sterile water. The embryos were put onto BAP medium [0.44% (*w*/*v*) MS powder (Sigma M5524), 0.075% (*w*/*v*) glutamine, 6% maltose (*w*/*v*), 1 μg/ml BAP (Sigma B9394) and 0.35% phytagel (*w*/*v*), pH 5.8]. After 6 weeks, the developing F_1_ plants were transferred to soil. Anthers from undeveloped spikes were transferred into a 0.7 M mannitol solution in petri dishes and incubated for 3 days at 24 °C. Then the anthers were transferred on BAP medium and incubated at 24 °C for 8 weeks in darkness. Developing calluses were transferred into containers containing MS sucrose medium (0.22% (*w*/*v*) MS, 1.5% (*w*/*v*) sucrose) and exposed to light for several days. Regenerating plants that turned green were planted into soil and left for seed formation in the greenhouse.

### Field tests

Field testing was done on the Danish island of Fyn during summers 2013 and 2014 using standard nitrogen fertilizer application for malting barley in plots of 7.5 m^2^ in size. 3600 grains were sown per experimental plot. All lines were tested in triplicates in randomized plots. The experimental plots were harvested using a combiner. The seeds were cleaned prior to weighing and the fraction above 2.2 mm was kept. The significance of differences in grain yield per plot was tested by a two sided t-test for two independent populations with equal variance assumption in Microsoft Excel.

### Germination test

All barley samples used in the manual malting experiments were evaluated for the parameters Germination index, Germination energy and Water sensitivity. Data was based on a sample size of 100 barley grains for a 4 ml or 8 ml germination test according to Analytica-EBC Method 3.6.2 (http://analytica-ebc.com/index.php?mod=contents&scat=10).

### Characterization of barley samples

Thousand kernel weights were determined by automatic counting using a Data Count JR (Data Technologies, Israel). Size fractionation within four classes (X > 2.8 mm), (2.8 mm < X > 2.5 mm), (2.5 mm < X > 2.2 mm) and (X < 2.2 mm) was done using a Pfeuffer Sortimat K3 (Pfeuffer GmbH, Germany). Size fractionation data was calculated from a 100 g sample. Protein, water and starch content of the barley samples were determined using a near infrared transmittance Foss 1241 NIT instrument (Foss, Denmark) and barley calibration Foss BY213271 provided by the manufacturer. 24 h prior to micro malting, the water content of the 100 g sample was re-determined using the same Foss 1241 NIT instrument but barley calibration Foss BY303300. All samples tested in micro malting experiments in this study were grown in rows in New Zealand from September 2012 to February 2013.

### Manual micro malting

All samples were malted using a manual micro malting system developed in house at the Carlsberg Research Laboratory. The micro malting was performed in a Termaks incubator KB8400 (Termaks AS, Norway) at 13.5 °C during the steeping and germination phase. The barley was malted in individual sample cups each holding 100 g of barley seeds. One batch of seeds was malted per line. The barley samples were steeped as a forced submission of all grains in a closed sample grid chamber for 3 h. The steeping procedure was performed in three 24 h intervals. Time recording started when grains were immersed in water first time and set to 0 h. The second steeping period started after 24 h and third after 48 h. The actual water uptake of the individual barley samples was estimated after a centrifugal removal of water from the sample. Steeping degree was calculated in relation to dry matter of the sample using a Foss 1241 NIT instrument (Foss A/S, Denmark) for determination of dry matter compared to the total sample weight of the sample after centrifugation. Near infrared transmittance is a non-destructive seed analysis, whereby a given chemical compound is assigned to a specific wavelength between 900 – 1050 nm. Based on a calibration of the instrument it is then possible predict the concentration of that given compound. Following the last steep the barley samples were maintained at a steeping degree at approximately 45% during the germination step. After the germination process the barley samples were kiln dried in the Termaks incubator for 21 h. The temperature profile of the kiln process was a two-step ramping profile and an isothermal terminal step monitored by a Eurotherm 815 control unit (Eurotherm by Schneider Electric, USA). First ramping step started at set point 30 °C and a linear ramping at 2 °C / h to the breakpoint at 55 °C. Second linear ramping was at 4 °C / h and reaching a maximum at 82 °C. This temperature was kept constant for 1.5 h. The process was based on a recirculating air flow using 80% fresh air. The kiln samples were cured using a manual root removal system from Wissenschaftliche Station für Brauerei, Munich, Germany. Final malt samples were stored at 20 °C prior to near infrared transmittance analysis and enzyme activity measurement.

### Malt analysis

Analysis of malt samples for water content, protein content, soluble protein and extract were determined with a Foss 1241 NIT (Foss A/S, Denmark) instrument using the Malt calibration Foss MA000010 provided by the manufacturer. Prior to enzyme activity analysis the malt samples were milled using a standard Foss Cyclotech mill (Foss A/S, Denmark) equipped with a tungsten carbide grinding ring (Foss 10,004,463), nickel plated impeller (Foss 1000 2666) and a 1 mm outlet screen (Foss 10,001,989). All enzyme activity measurements of barley malt were made within 48 h after milling of the dry sample.

### α-amylase activity

α-amylase activity of final malt was based on malt flour made according to the sample preparation description in the Malt Analysis section above. α-amylase activity assays were measured using a Ceralpha kit (K-CERA) from Megazyme (Ireland) using standard laboratory equipment. The amylase assays were made according to the manufacturer’s protocol (K-CERA 01/12). Measurements were made in technical duplicates. Calculation of amylase activity was based on the formula in the Megazyme protocol (K-CERA 01/12). The significance of differences in α-amylase activity was tested by a two sided t-test for two independent populations with equal variance assumption in Microsoft Excel comparing the four double haploid lines carrying the *brh1.a* allele to the three lines in their progeny, BW074, Bowman and Quench.

### β-amylase activity

β-amylase activity of final malt was based on malt flour made according to the sample preparation description in the Malt Analysis section above. β-amylase activity assays were measured using Betamyl kit (K-BETA3) from Megazyme (Ireland) using standard laboratory equipment. The amylase assays were made according to the manufacturer’s protocol (K-BETA3 10/10). Measurements were made in technical duplicates. Calculation of β-amylase activity was based on the formula in the Megazyme protocol (K-BETA3 10/10). The significance of differences in β-amylase activity was tested by a two sided t-test for two independent populations with equal variance assumption in Microsoft Excel comparing the four double haploid lines carrying the *brh1.a* allele to the three lines in their progeny, BW074, Bowman and Quench.

### Limit dextrinase activity

Limit dextrinase activity of final malt was based on malt flour made according to the sample preparation description in the Malt Analysis section above. Limit dextrinase activity assays were measured using a Limit Dextrizyme kit T-LDZ1000 from Megazyme (Ireland) using standard laboratory equipment. The limit dextrinase assays were made according to manufacturer’s protocol T-LDZ1000 07/9. Measurements were made in technical duplicates. Calculation of limit dextrinase activity was based on the formula in the Megazyme protocol T-LDZ1000 07/9. The significance of differences in Limit dextrinase activity was tested by a two sided t-test for two independent populations with equal variance assumption in Microsoft Excel comparing the four double haploid lines carrying the *brh1.a* allele to the three lines in their progeny, BW074, Bowman and Quench.

### Public databases

Public sequence databases were accessed via the following web pages: “http://www.gramene.org/” for sorghum, “http://rice.plantbiology.msu.edu/analyses_search_blast.shtml” for rice, and “http://brachypodium.org/” for brachypodium. The NCBI probe database was accessed via the link http://www.ncbi.nlm.nih.gov/probe. The HarvEST database was accessed using the link http://harvest.ucr.edu/.

### Screening of a pooled BAC library of the barley genome

Bacterial artificial chromosome (BAC) clones originating from six independent BAC libraries were previously made from the barley cultivar Morex [27] and pooled in multidimensional levels [2]. Primers specific for the *Brh1* target region (Table 2) were obtained from Eurofins MWG Operon, Ebersberg (Germany) and were used for a PCR screening of the pooled BAC libraries to identify BAC clones containing the region surrounding the *Brh1* gene.

**Table 2:** Oligonucleotides used as PCR primers to amplify and sequence the gene encoding the Gα-subunit of the heterotrimeric G protein and to amplify gene fragments located in the genomic region surrounding this gene in a BAC pool screening

Primer name	Sequence (5′ → 3′)	Template	Length of amplification product [bp]
bw-Fr1-F1	TGTCAAGATGATGGCTTCAG	Genomic DNA	703 (with bw-Fr1-R1)
bw_Fr1-Fseq	AAAAAGACGATGATGAAGC	Genomic DNA	
bw-Fr1-R1	GATCGACGAGAGCATGAGAC	Genomic DNA	
bw-Fr2-F1	GGCGAGGTAGAAAGCAAAAG	Genomic DNA	790 (with bw-Fr2-R1)
bw-Fr2-R1	TTTTGAGGTATCCATCCATCTC	Genomic DNA	
bw-Fr3-F1	CGCACACAGTCAAAGGAAC	Genomic DNA	896 (with bw-Fr3-R1)
bw-Fr3-R1	GACAGCATTGCACAAGGAG	Genomic DNA	
bw-Fr4-F1	TGTCCCAGATCCTCAAACTG	Genomic DNA	901 (with bw-Fr4-R1)
bw-Fr4-R1	TTGTCCCTTGTAAACTGTTGG	Genomic DNA	
bw-Fr5-F1	TCCTCCTGCAAAATCTCTCC	Genomic DNA	903 (with bw-Fr5-R1)
bw-Fr5-R1	TTAGACTCGGCATTTTGAGG	Genomic DNA	
bw-Fr6-F1	ACCTGAATGGCTGGATCTTC	Genomic DNA	857 (with bw-Fr6-R1)
bw-Fr6-R1	GCATAGTGGGGATTATTCAGG	Genomic DNA	
bw-Fr7-F1	GGTCCAAACAGGTTCAGTTG	Genomic DNA	824 (with bw-Fr7-R1)
bw-Fr7-R1	GATTCTGCACGAGACAAAGG	Genomic DNA	
bw-Fr8-F1	GGAATCAGTCTTTCCAGATCC	Genomic DNA	705 (with bw-Fr8-R2)
bw-Fr8-R2	CCAAAATACCCATACCAACC	Genomic DNA	
bw-Fr9-F1	GAGCGAGCCAGAGATTTTG	Genomic DNA	711 (with bw-Fr9-R1)
bw-Fr9-R1	TGTATATGACGGAGCAGCAAG	Genomic DNA	
bw_Fr9-Rseq	GCTTGTTGGTTGTAGCTCAG	Genomic DNA	
IL130	CAACAATATGGGCATTACAT	BAC	350 (with IL131)
IL131	CGAGGAGTTCTACAAATCAT	BAC	
IL132	CTTGCCCACTTACCAGGTGG	BAC	300 (with IL133)
IL133	ACGGGATCTCCTTATGGAGC	BAC	
IL134	ATGATCGACTGCTACATCC	BAC	318 (with IL135)
IL135	GGTTCTGTCGTTGTACCTAG	BAC	
IL136	CACCAAGCAGCAGATGATC	BAC	152 (with IL137)
IL137	CCAGTTTGTTGGATGTTTCC	BAC	
IL138	GAGGGCTTCTATTGAAAGTG	BAC	510 (with IL139)
IL139	GGTACTGACCAACTGTTACG	BAC	
IL140	AAAAGGCTGACTCAAAGACC	BAC	318 (with IL141)
IL141	CGACAAGCTCCATAAGGTAC	BAC	
IL142	TCTGACCCAGAAACTTGATC	BAC	651 (with IL143)
IL143	GAAAGGGAGAGAGAAATCG	BAC	
IL144	GGTCTGACCTCTTATTTCCC	BAC	512 (with IL145)
IL145	AAAGCGAGGCATTCACTAG	BAC	
IL146	GCTCAAAGTGGGAATACGAG	BAC	475 (with IL147)
IL147	CTTGTGCTGCCAACTAGATG	BAC	
IL148	AAGAGCATCCAGTGACAAG	BAC	633 (with IL149)
IL149	GGATCTCCGCTTAGGTATAG	BAC	
IL150	CGCTTTCCCTATTTTGTCAG	BAC	576 (with IL151)
IL151	ACTTCACGGAGATTGATGC	BAC	
IL152	AGCCAGCAATCCTTGTTAGC	BAC	510 (with IL153)
IL153	ACCTTGAGCCAGGGAAAAG	BAC	
IL201	CTCTGGCTCGCTCATCAACC	RNA	1229 (with IL202)
IL202	AGCGTCTCATGCTCTCGTCG	RNA	
IL203	GTAAACGAGGCAGAGGCAGC	RNA	461 (with IL204)
IL204	CAGTCGGGAACTTGCAGCAC	RNA	
IL205	CGGAACTCAAGGGCTACACG	RNA	600 (with IL206)
IL206	CGAACAGCTCCTTGGTCTCC	RNA	
IL207	AAATCTCTGGCTCGCTCATC	RNA	1389 (with IL208)
IL208	AGCACAAAGGAGTCACAAGG	RNA	
IL209	ACTAGGAGGAAGCATGAGAC	RNA	1305 (with IL210)
IL210	CAATACAGAGTTGCGACACC	RNA	

### PCR amplification and sequencing of the Gα-subunit of the heterotrimeric G protein in barley

Oligonucleotides used for PCR amplification and DNA sequencing of the Gα-subunit of the heterotrimeric G protein are listed in Table 2. PCR amplification from genomic DNA and sequencing of PCR products was done at LGC Genomics GmbH (Berlin, Germany) in the case of all *brh1* original mutant lines, near-isogenic lines and the respective mother cultivars. In the case of *ari-m* mutant lines and their respective mother cultivars, isolation of genomic DNA and PCR amplification was done using the REDExtract-N-Amp™ Plant PCR Kit (Sigma-Aldrich, St. Louis, USA), and sequencing of PCR products was done at StarSEQ (Mainz, Germany).

RNA was isolated from seedlings leaves with the help of Trizol reagent (Thermo Fisher Scientific, Waltham, USA). For one-step RT-PCR of RNA the SuperscriptIII one-step RT-PCR kit (Thermo Fisher Scientific, Waltham, USA) with HIFI taq enzyme was used. Sequencing of PCR products was done at StarSEQ (Mainz, Germany).

### Allelism crosses to confirm identity of the *Ari-m* and the *Brh1* locus in barley

Allelism tests were performed through crosses between BW074 (*brh1.a*) and BW051 (*ari-m.28*). Plants were grown in a greenhouse at 18°C under 16-h-light / 8-h-dark cycles. Light intensity were set to a photon flux of 300 μmol m^−2^ s^−1^. Plants were pollinated 3 days after emasculation. F_1_ progenies of this cross were genotyped to confirm successful crossings, and grown to maturity for phenotypic analysis to confirm allelism through visual inspection of plants.

## Results

### Identification of the *Brh1* locus

**Fig. 2:**
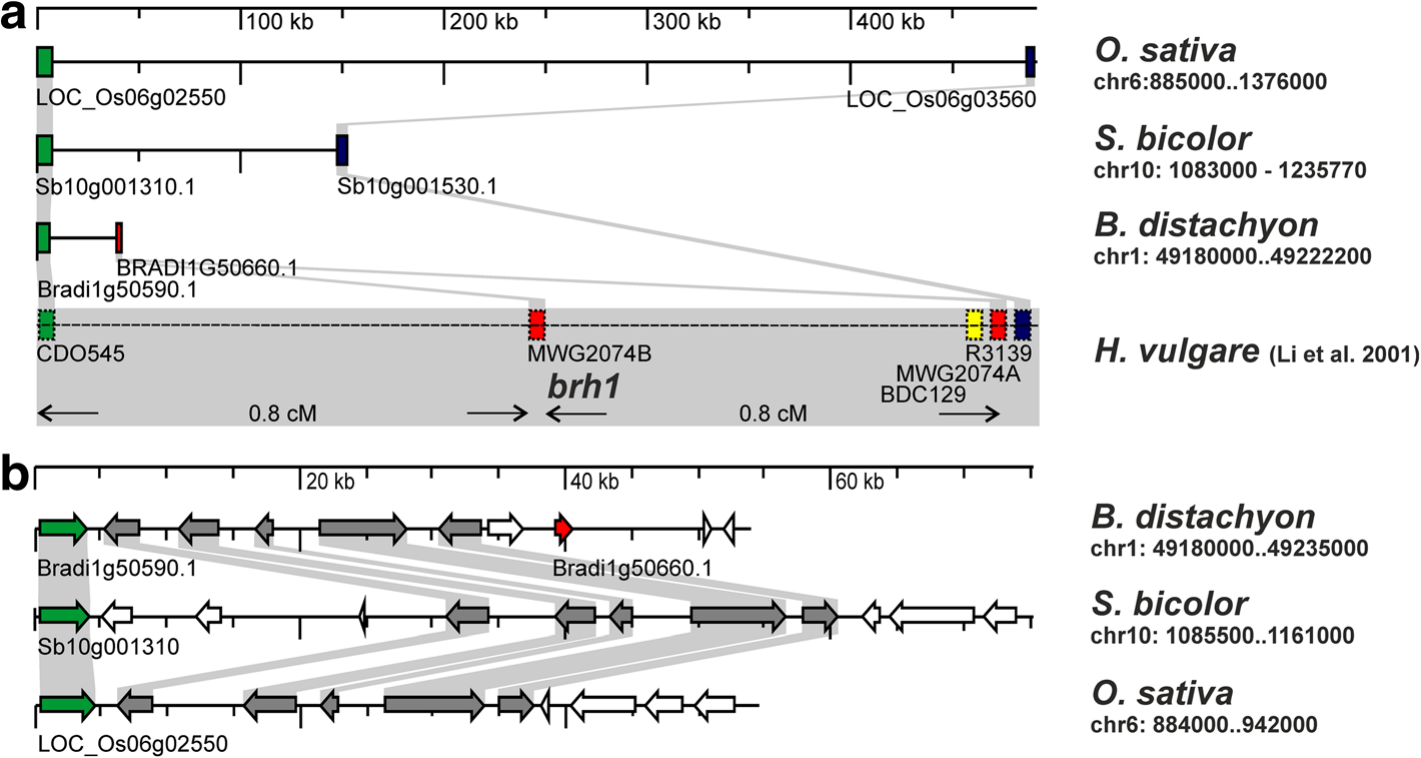
The *Brh1* genomic region in barley and the syntenic regions in rice, sorghum and brachypodium. **a**. *Brh1* has been mapped between markers CDO545 (green, NCBI accession numbers AA231869.1 and AA231713.1) and BDC129 (yellow), R3139 (blue, AU082160, D25093) and MWG2074 (red, AJ234754.1, AJ234755.1) [21]. MWG2074 was shown to have two loci, the B locus (middle, red) was co-segregating with *Brh1*. Marker sequences were used to identify matching loci in rice, sorghum and brachypodium (color codes kept). No obvious hits to orthologous sequences could be found for marker MWG2074 in rice and sorghum, or for R3139 in brachypodium. **b**. Gene content of the *Brh1* syntenic region in brachypodium as identified in Fig. 2a. The loci (Bradi1g50590 and Bradi1g50660) matching markers CDO545 and MWG2074 are highlighted green and red, respectively. The genes flanked by loci Bradi1g50590 and Bradi1g50660 are found in the same order in rice and sorghum. The orthologous barley genes were used to screen pooled BAC libraries

The *Brh1* gene is located on barley chromosome 7HS, flanked by markers CDO545 (distal) and R3139 (centromeric) [21]. The two markers are both located 0.8 cM away from the *Brh1* locus. A third marker, MWG2074, was shown to co-segregate with the *brh1* phenotype in the considered mapping population [21]. Due to the close proximity of the three markers to each other and *Brh1*, we decided to sequence their neighboring genes in all *brh1* mutants in order to identify the *Brh1* gene. The work was initiated before barley genomic sequence information was publically available [22]. Therefore, the marker sequences were downloaded from the NCBI probe database and used for blastn analysis of genomic databases. As a first step, sequences and genes homologous to the three barley markers of interest were identified in the genomes of rice (*Oryza sativa*), sorghum (*Sorghum bicolor*) and brachypodium (*Brachypodium distachyon*) (Table 3). We postulated that a syntenic approach could help us to visualize barley genes in the region. However, the comparison showed that the gene content of the *Brh1* target region is not fully conserved among the different grass genomes as not all markers flanking the *Brh1* locus in barley could be identified in close proximity to each other in any of the rice, sorghum and brachypodium genomes (Fig. 2). The two markers CDO545 and MWG2074, which are flanking the *Brh1* locus in barley, were found to be located close to each other on chromosome 1 in brachypodium. Further, six genes were present in the region of interest in all three organisms investigated (green and gray arrows in Fig. 2b). In case of five of these genes, a unigene could be identified in the barley EST database HarvEST (http://harvest.ucr.edu/). Primers were designed for these unigenes and a PCR-based screening of pooled BAC libraries was performed. We identified ten BAC clones, located in two finger-print contigs on chromosome 7H. The two finger-print contigs, 44,369 and 44,201, were both anchored to the genomic position at 12.74 cM on chromosome 7H (17). The BAC clones identified in the PCR-screen were overlapping with three sequenced BAC clones (17), providing us with a partial sequence of the *Brh1* target region in barley. The six genes predicted to be located in this region could all be identified on the sequenced BACs. The three sequenced BAC clones were searched for appropriate candidate genes for the *Brh1* mutation. On BAC clone HVVMRX83KHA0060C10 we could identify the Morex locus MLOC_67224.3, a sequence corresponding to the NCBI accession AF267485. This gene encodes the Gα subunit of a heterotrimeric G protein in barley. Mutations in the Gα subunit are known to be the cause of the semi-dwarf phenotype in the *dwarf1* (*d1*) rice mutants [4], making the Gα subunit a promising candidate gene for the *brh1* mutation in barley. Still, the location of the barley gene was a surprising finding. The *D1* gene in rice (locus Os05g26890) is located on chromosome 5 and not in the region on chromosome 6, which was shown to be syntenic to the *Brh1* region in barley [21]. Further, blastp analyses identified Sb01g045320 in sorghum and Bradi2G60350 in brachypodium as the orthologous genes of barley *Brh1*. Neither of the sorghum and brachypodium genes are in regions syntenic to barley.

**Table 3:** Markers in the vicinity of *Brh1* [21] and the gene models associated with the best blastn hit in the genomes of rice, sorghum and brachypodium

Marker	NCBI accession no.	Rice blast hit	Sorghum blast hit	Brachypodium blast hit
CDO545	AA231869.1 AA231713.1	Os06g02550.2	Sb10g001310	Bradi1g50590.1
MWG2074	AJ234755.1 AJ234754.1	no hit on chromosome 6	no hit on chromosome 10	Bradi1g50650.1 Bradi1g50660.1
R3139	AU082160 D25093	Os06g03560	Sb10g001530 (with D25093)	no conclusive hit

### *Brh1* encodes the Gα subunit in barley

**Fig. 3:**
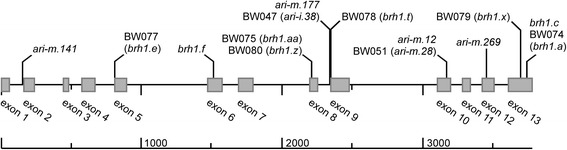
Distribution of mutations identified in the gene of the Gα subunit of the heterotrimeric G protein in *brh1*, *ari-i* and *ari-m* barley mutants. The gene contains 13 exons and 12 introns over 3780 bp. The deduced polypeptide is 383 amino-acid residues. More detailed information about the mutations is found in Table 4. Mutations *brh1.ae* and *ari-m.251* are large deletions of >3 kb not shown in the figure

Mutations in the gene encoding the Gα subunit in rice cause the *d1* mutant phenotype, a semi-dwarf phenotype similar to the *brh1* phenotype in barley. Rice *d1* mutants are characterized by reduced plant height, shorter panicles, and shorter, globe-shaped seeds [4]. The barley *brh1* mutation also causes height reduction, changes in the spike architecture and is characterized by a rounded seed shape (Fig. 1). The matching rice phenotype and the localization of the Gα gene in the *brh1* target region made the gene encoding the Gα subunit an obvious candidate gene for the *brh1* mutation. Therefore, we sequenced the gene in the *brh1* near-isogenic lines and in the cultivar Bowman. In each of the eight near-isogenic lines, but not in Bowman, a mutation was found with strong postulated impact on the resulting protein (Fig. 3, Table 4). In BW076 (*brh1.ae*) only two of the nine PCR fragments that were generated for sequencing of the *Brh1* gene could be amplified from genomic DNA suggesting a partial deletion of the gene in this mutant line. Small deletions of 1 or 2 bp caused frame shifts in BW047 (*ari-i.38*), BW075 (*brh1.aa*), BW077 (*brh1.e*), BW078 (*brh1.t*) and BW080 (*brh1.z*). The frame shifts introduced premature stop codons in all lines except BW074 (*brh1.a*) where a new stop codon was introduced in the 3′ untranslated region after a frame shift, thus extending exon 13. In BW079 (*brh1.x*) a point mutation introduced a premature stop deleting the last 20 amino acids of the protein.

**Table 4:** Mutations identified in recessive *brh1* alleles and their deduced effect at protein level

Allele	Position of mutation^a^	Type of mutation	Effect of mutation on protein
BW047 (*ari-i.38*)	2345	a to t	Mutation shifts splice site between intron 8 and exon 9 leading to frame shift. 203 native amino-acid residues followed by FSAAPLERAKEAERYTGCMM.
BW074 (*brh1.a*)	3745-3746	2 bp deletion	Frame shift eliminates wild type stop codon and extends exon 13. 372 native residues followed by 152 additional aa residues.
BW075 (*brh1.aa*)	2205	1 bp deletion	Frame shift in exon 8. 185 native residues followed by MCSMQEYGQMGL.
BW076 (*brh1.ae*)	Deletion (minimal region: −723 to 2819	Large deletion, no transcript detectable	Probably no protein produced
*brh1.c*	3745-3746	2 bp deletion	Frame shift eliminates wild type stop codon and extends exon 13. 372 native residues followed by 152 additional residues.
BW077 (*brh1.e*)	813	g to a	Mutation eliminates splice site between intron 4 and exon 5 leading to frameshift. 88 native residues followed by VCYYWKGCVSYLFPHLLHSGDYSGNLHWIFLWECVYTSYY.
*brh1.*f	1512-1515	4 bp deletion	Frame shift and premature stop in exon 6. 129 native residues followed by SLIKNSYRM.
BW078 (*brh1.t*)	2346	g to a	Mutation shifts splice site between intron 8 and exon 9. 203 native residues followed by FSATPLERAKEVERYTGCMM.
BW079 (*brh1.x*)	3718	a to t	Premature stop in exon 13. Truncated protein of 363 residues.
BW080 (*brh1.z*)	2205	1 bp deletion	Frame shift in exon 8. 185 native residues followed by MCSMQEYGQMGL.
*ari-m.12*	3178	g to a	Premature stop in exon 10. Truncated protein of 270 native residues.
BW051 (*ari-m.28*)	3178	g to a	Premature stop in exon 10. Truncated protein of 270 native residues.
*ari-m.141*	164	g to a	Mutation eliminates splice site between intron 1 and exon 2. 21 native residues followed by VSSFPYHLLDSFNSSCPVLSCPVLS.
*ari-m.177*	2345	a to t	Mutation shifts splice site between intron 8 and exon 9 leading to frame shift. 203 native residues followed by FSAAPLERAKEAERYTGCMM.
*ari-m.251*		Large deletion	No protein produced.
*ari-m.269*	3449	g to a	Premature stop in exon 12. Truncated protein of 306 residues.

The deduced polypeptide of *Brh1* (AF267485) is 383 amino-acid residues

^a^First position is the “A” of the ATG start codon in the Bowman genomic sequence

**Fig. 4:**
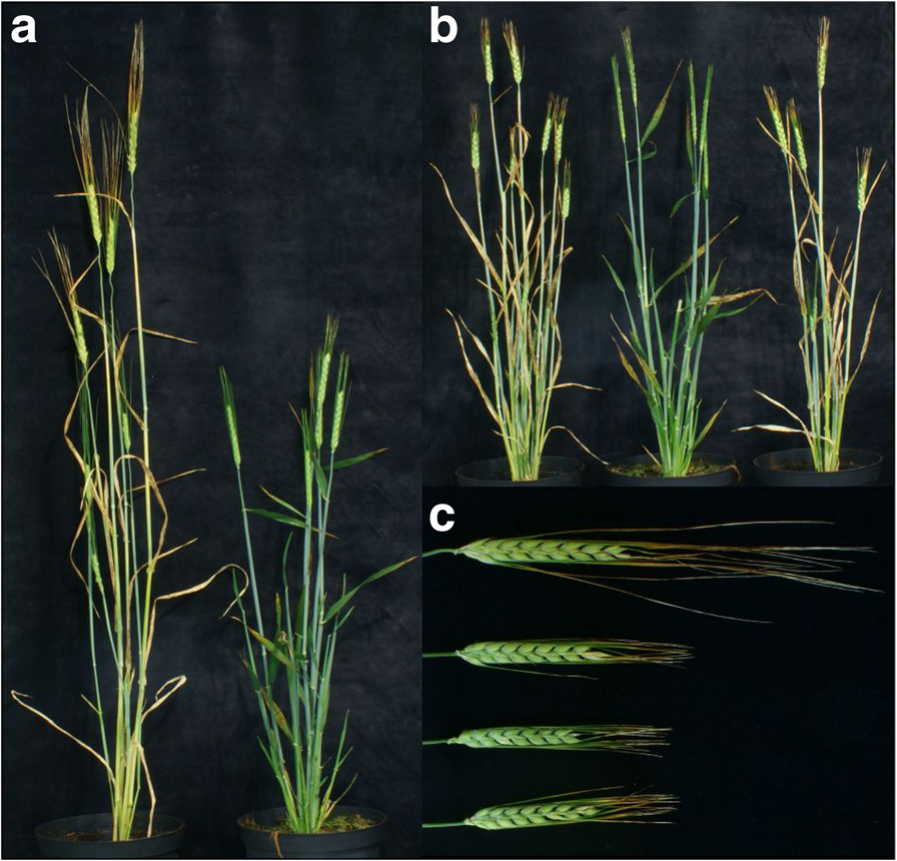
Phenotype of F_1_ plants generated from a cross between the Bowman near-isogenic lines BW051 (*ari-m.28*) and BW074 (*brh1.a*). **a**. Overall height of cultivar Bowman (left) compared to an F_1_ plant of a cross between BW051 x BW074. **b**. Overall height of BW051 x BW074 F_1_ plant (middle), BW051 (left) and BW074 (right), carrying the recessive *ari-m.28* and *brh1.a* alleles, respectively. **c**. Awn phenotypes of Bowman (top), BW051, F_1_ of BW051 x BW074 and BW074

**Fig. 5:**
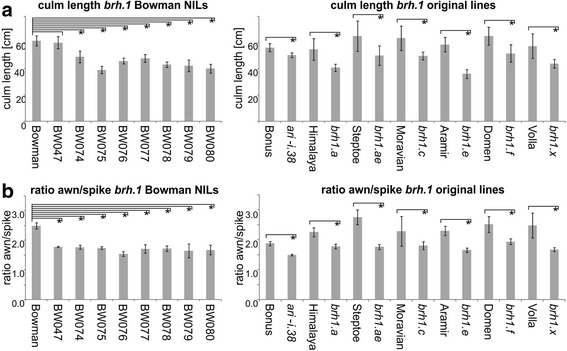
Culm length (**a**) and awn/spike ratio (**b**) in *brh1* mutants compared to their respective mother cultivars. The plants were grown in a green house. A *p*-value of <0.05 was considered as significant and indicated with an asterisk in the figure

**Fig. 6:**
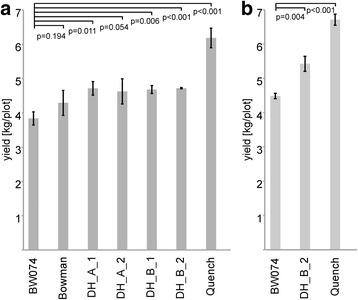
Grain yield in field trials. Near-isogenic line BW074 (*brh1.a*) and two double-haploid lines obtained from a cross of cultivar Quench x BW074 are compared to Quench and Bowman. The field trials were performed on the Danish island of Fyn in the summer 2013 (**a**) and 2014 (**b**). All represented lines were tested in triplicates. Significance was tested in relation to BW074. DH_A_1, DH_A_2, DH_B_1, and DH_B_2 designate double-haploid lines obtained from a cross of Quench x BW074. Lines A and B depict two independent double-haploid lines regenerated after tissue culture, 1 and 2 relates to separate propagations of the same line. A *p*-value of <0.05 was considered as significant

**Fig. 7:**
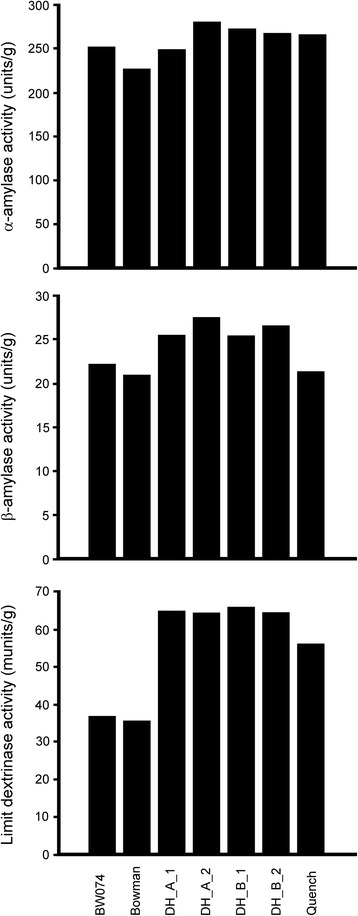
Measurement of α-amylase, β-amylase and free limit dextrinase activity in malt samples obtained from near-isogenic line BW074 (*brh1.a*), two double-haploid lines obtained from a cross of cultivar Quench x BW074, and of cultivars Quench and Bowman. Two measurements per sample were performed. Standard deviations are indicated in the figure. The averages of the activities of the double-haploid lines were compared to the average of the activities of BW074, Bowman and Quench in a t-test, which revealed significant differences in enzyme activities for β-amylase and free limit dextrinase (*p* < 0.05)

We also sequenced the gene encoding the Gα subunit from the original mutants and two additional lines, *brh1.c* and *brh1.f*, for which no near-isogenic lines have been generated (Fig. 3, Table 4). All mutations were verified and they were not present in the respective mother cultivar (Table 1). Mutation *brh1.c* was based on a 2 bp deletion and identical to *brh1.a*. Mutation *brh1.f* was based on a 4 bp deletion in exon 6 leading to a premature stop. We noticed several SNPs between Bowman and the mother cultivars (Bonus, Himalaya, Steptoe, Moravian, Aramir, Domen, Akashinriki and Volla) with the exception of Aapo, which was identical to Bowman over the sequenced region (Additional file 1 Table S1). We noted that the SNP pattern in mutant *brh1.c* was not the same as its mother cultivar Moravian. Instead they were identical to those of *brh1.a* and Himalaya. Still mature plants of the mutant lines *brh1.c* and *brh1.a* show different morphological characters – *brh1.c* is two-rowed, while *brh1.a* is six-rowed. We therefore suggest that the *brh1* mutation was introduced into Moravian by crossing to a *brh1.a* mutant line instead of an independent mutational event.

### Identification of additional *brh1* alleles

When propagating various near-isogenic lines, we noticed that BW051 (*ari-m.28*) has a *brh1*-like phenotype with overall reduction in height and characteristic short awns. In addition, the introgression residing from the original mutant donor *ari-m.28* in BW051 is reduced to a minor genetic element in the telomeric region of chromosome 7HS overlapping with the introgression regions of all *brh1* Bowman near-isogenic lines (Table 1, Table 5). When sequencing the gene encoding the Gα subunit in BW051 we found a single nucleotide substitution in exon 10 leading to a premature stop codon (Fig. 3, Table 4). The same mutation was found in the original mutant line *ari-m.28*, which was isolated in the cultivar Bonus [20]. We also performed a cross between BW074 (*brh1.a*) and BW051 (*ari-m.28*). The F_1_ plants displayed a semi-dwarf and short-awn mutant phenotype, which further proves that *ari-m.28* and *brh1.a* are allelic (Fig. 4).

**Table 5:** Summary of *breviaristatum-m* (*ari-m*) alleles, their genetic backgrounds, their availability as Bowman near-isogenic lines, and introgression regions on chromosome 7H

Allele	Genetic background	Bowman near-isogenic line	Introgression region on 7HS (cM)
*ari-m.12* ^*a*^	Bonus	no line available	-
*ari-m.28* ^*a*^	Bonus	BW051^a^	4.74 – 9.55
*ari-m.141* ^*a*^	Foma	no line available	-
*ari-m.177* ^*a*^	Foma	no line available	-
*ari-m.251* ^*a*^	Kristina	no line available	-
*ari-m.269* ^*a*^	Kristina	no line available	-

The near-isogenic line has been prepared previously as well as the determination of the introgression region [11]

^a^Source of seed material: Nordic Genetic Resource Center, Alnarp, Sweden

In addition to *ari-m.28*, five other *ari-m* alleles are available (Table 5) (www.nordgen.org/bgs). A mutation in the gene encoding the Gα subunit could be identified in each of them (Fig. 3, Table 4). The mutations found in *ari-m.12* and *ari-m.28* are identical nucleotide substitutions introducing stop codons in exon 10. Mutant line *ari-m.177* was identical to *ari-i.38* (BW047), affecting a splice site between intron 8 and exon 9. Line *ari-m.141* also showed a mutation in a splice site – between intron 1 and exon 2. Mutation *ari-m.269* is a nucleotide substitution introducing a stop codons in exon 12. Finally, *ari-m.251* is a large deletion since no parts of the *Brh1* locus could be amplified by PCR.

### Transfer of the *brh1* mutation to a modern elite malting barley background

The available *brh1* mutant alleles have been generated in several genetically different mother cultivars. In our trials, they all showed a pronounced reduction in culm length and awn length (Fig. 5). To the best of our knowledge, none of the described *brh1* alleles have been tested in European breeding material. Therefore, we performed crosses between the Bowman near-isogenic line BW074 (*brh1.a*) and the European elite malting cultivar Quench. Double-haploid plants were generated. Two independent double-haploid lines, designated DH_A and DH_B that displayed the phenotype characteristic for *brh1* mutant lines, were selected. These lines were propagated and finally tested under field conditions in two subsequent years (Fig. 6). It was observed that cultivar Bowman produced significantly lower grain yield than cultivar Quench. This was expected, as Bowman is a North American cultivar while Quench was bred for agriculture in Europe. Mutant line BW074 had a yield comparable to that of Bowman. The double-haploid lines yielded higher than Bowman and BW074 but lower than Quench. A repetition of the field trial with a reduced number of lines in the following year gave similar results (Fig. 6b). We conclude that the yield of *brh1* mutant lines can be significantly increased by backcrossing to a modern elite line.

### The effect of the *brh1* mutation on malting quality

The *brh1* double-haploid lines generated in this study were tested in laboratory scale micro malting experiments together with the near-isogenic line BW074 (*brh1.a*) and the cultivars Quench and Bowman. We measured the activity of three key starch degrading enzymes α-amylase, β-amylase and free limit dextrinase (Fig. 7). The *brh1* double-haploid lines showed an increase in activity, especially for β-amylase and free limit dextrinase activity. The reason for this observation remains elusive but can partly be caused by a changed volume-area ratio in the global shaped *brh1* seeds. Still, the presented results do not indicate that the *brh1* mutation has a negative effect on malting quality in barley.

## Discussion

While there are several genes encoding Gα, Gβ and Gγ subunits of heterotrimeric G proteins in mammals, there is only one Gα, one Gβ and three Gγ encoding genes in arabidopsis, and one Gα, one Gβ and four Gγ genes in rice [29]. Analysis of the recently sequenced barley genome [22, 17]) reveals one Gα (gene identity in Mascher et al. [22] HORVU7Hr1G008720.11), one Gβ (HORVU4Hr1G003110.4) and four Gγ genes (HORVU5Hr1G093530.3, HORVU5Hr1G093550.1, AK367089, HORVU5Hr1G061840.7). In mammalian systems, binding of a ligand to the G-protein-coupled receptor causes a rapid exchange of GDP to GTP bound by the Gα-subunit, which promotes a dissociation of activated Gα-GTP from the Gβγ heterodimer [32]. Activated Gα-GTP and/or Gβγ interacts with effector proteins. A different system for G-protein signaling is found in plants. The arabidopsis Gα subunit spontaneously binds GTP in vitro [19] but its ability to stimulate effector molecules is inhibited by its association to a membrane bound Regulator-of-G-protein-signaling (RGS) protein [34]. Upon binding of a ligand to the RGS protein, the Gα-GTP subunit is released and can interact with effector molecules.

In the present study we demonstrated that barley *Brh1* is orthologous to rice *D1* encoding a Gα-subunit of a heterotrimeric G protein [4]. In addition, we characterized nine *brh1*, one *ari-i* and six *ari-m* mutants at the DNA level, and demonstrate that *Ari-m* is allelic to *Brh1*. It has previously been reported that *Brh1* and *Ari-i* are allelic [33]. All sixteen mutations result in severe changes of the *Brh1* gene and gene product – deletions, nonsense mutations or frame shifts leading to modified polypeptides. It was recently shown that the barley *Ari-e* encodes a Gγ subunit of a heterotrimeric G protein [18, 35], being orthologous to *DEP1* in rice [16]. Further, the gene product of *TUD1* in rice, a U-box E3 ubiquitin ligase, has been suggested to directly interact with the rice Gα subunit and is therefore directly responsible for the turnover of heterotrimeric G proteins [15]. We found recently that barley *Brh2* is orthologous to *TUD1* in rice (Braumann et al. unpublished). Barley *brh1*, *brh2* and *ari-e* mutants have short-culm phenotypes, which is now understood in the light of their connection to the heterotrimeric G protein.

Although genes encoding subunits of the heterotrimeric G protein complex now start to be identified, it is still not clear in which signaling pathway the complex is participating. The response to both brassinosteroids and gibberellic acids have been investigated for *brh1* mutants in previous studies. In contrast to *uzu* mutants, *brh1* mutants are brassinosteroid sensitive [14], making it unlikely that *brh1* is part of the brassinosteroid signaling pathway. It has further been shown that application of gibberellic acid leads to increased elongation of *brh1* plants in a similar extend as for wild type plants. Still, both gibberellic acid treated and untreated *brh1* plants are significantly shorter than wild type plants that had received the same treatment [5]. These findings disprove the direct involvement of the *Brh1* gene in either gibberellic acid signaling or biosynthesis.

Dahleen et al. [8] did a detailed study of the agronomic performance of five recessive *brh1* lines – BW047 (*ari-i.38*), BW074 (*brh1.a*), BW078 (*brh1.t*), BW080 (*brh1.z*) and BW075 (*brh1.aa*). The parameters including plant height, lodging, yield, number of kernels per spike and kernel weight. All *brh1* plants were shown to be significantly shorter and more lodging resistant than the cultivar Bowman. The mutants displayed a lower kernel weight but otherwise yields were like Bowman with the exception of BW075 (*bri1.aa*), which yielded lower. Based on their results it was concluded that the *brh1* mutations introduce agronomically valuable traits like shorter plant stature and lower lodging potential while negative effects of the mutation on agronomical key traits like yield seem to be neglectable [8]. Our study indicated a lower yield due to the *brh1* mutation but we assume that recurrent backcrossing to Quench would increase the yield like it did in the Bowman near-isogenic lines studied by Dahleen et al. [8]. More important, our results showed no negative effect on malting qualities due to the *brh1* mutation. We therefore suggest that *Brh1* might be a competitive breeding alternative to other used semi-dwarfing genes like *Ari-e*, *Ert-k*, *Sdw1*, *Sdw4* and *Uzu1*.

## Conclusions

Mutants in the *Brh1* locus are deficient in the Gα subunit of a heterotrimeric G protein, which shows that heterotrimeric G proteins are important regulators of culm length in barley. It is still not clear in which signaling pathway the complex is participating. Sixteen mutant alleles were characterized at DNA level and display a repertoire of deletions and nucleotide substitutions. While our study suggests a lower yield due to mutations in *Brh1*, we did not see any negative effects on malting quality. Thus, *brh1* mutant alleles might be used in order to breed for robust and lodging resistant malting barley cultivars.

## Additional file

Additional file 1: Table S1. SNP and mutation information for Brh1 mutant lines and original background cultivars. (XLSX 121 kb)
